# Potential of Magnetic Nanofiber Scaffolds with Mechanical and Biological Properties Applicable for Bone Regeneration

**DOI:** 10.1371/journal.pone.0091584

**Published:** 2014-04-04

**Authors:** Rajendra K. Singh, Kapil D. Patel, Jae Ho Lee, Eun-Jung Lee, Joong-Hyun Kim, Tae-Hyun Kim, Hae-Won Kim

**Affiliations:** 1 Institute of Tissue Regeneration Engineering (ITREN), Dankook University, Cheonan, South Korea; 2 Department of Nanobiomedical Science and BK21 Plus NBM Global Research Center for Regenerative Medicine, Dankook University, Cheonan, South Korea; 3 Department of Biomaterials Science, College of Dentistry, Dankook University, Cheonan, South Korea; University of Milan-Bicocca, Italy

## Abstract

Magnetic nanofibrous scaffolds of poly(caprolactone) (PCL) incorporating magnetic nanoparticles (MNP) were produced, and their effects on physico-chemical, mechanical and biological properties were extensively addressed to find efficacy for bone regeneration purpose. MNPs 12 nm in diameter were citrated and evenly distributed in PCL solutions up to 20% and then were electrospun into nonwoven nanofibrous webs. Incorporation of MNPs greatly improved the hydrophilicity of the nanofibers. Tensile mechanical properties of the nanofibers (tensile strength, yield strength, elastic modulus and elongation) were significantly enhanced with the addition of MNPs up to 15%. In particular, the tensile strength increase was as high as ∼25 MPa at 15% MNPs vs. ∼10 MPa in pure PCL. PCL-MNP nanofibers exhibited magnetic behaviors, with a high saturation point and hysteresis loop area, which increased gradually with MNP content. The incorporation of MNPs substantially increased the degradation of the nanofibers, with a weight loss of ∼20% in pure PCL, ∼45% in 10% MNPs and ∼60% in 20% MNPs. Apatite forming ability of the nanofibers tested *in vitro* in simulated body fluid confirmed the substantial improvement gained by the addition of MNPs. Osteoblastic cells favored the MNPs-incorporated nanofibers with significantly improved initial cell adhesion and subsequent penetration through the nanofibers, compared to pure PCL. Alkaline phosphatase activity and expression of genes associated with bone (collagen I, osteopontin and bone sialoprotein) were significantly up-regulated in cells cultured on PCL-MNP nanofibers than those on pure PCL. PCL-MNP nanofibers subcutaneously implanted in rats exhibited minimal adverse tissue reactions, while inducing substantial neoblood vessel formation, which however, greatly limited in pure PCL. *In vivo* study in radial segmental defects also signified the bone regeneration ability of the PCL-MNP nanofibrous scaffolds. The magnetic, bone-bioactive, mechanical, cellular and tissue attributes of MNP-incorporated PCL nanofibers make them promising candidate scaffolds for bone regeneration.

## Introduction

Scaffolds for tissue engineering play important roles in providing sites for anchorage of stem/progenitor cells and their self-renewal and possible lineage specific differentiation [1]–[3]. Designing scaffolds to be nontoxic with a proper degree of degradability and to have active actions involved in tissue repair and regenerative processes are considered primary requirements [4]–[6]. Biochemical cues provided by the scaffolds such as surface chemistry and release of soluble factors have been most widely documented. Biophysical factors including surface roughness, topology, and matrix elasticity have also been recently researched to determine the behaviors of cells, particularly stem cells, and their ultimate potential for tissue engineering [7]–[9].

One interesting cue that has been recently proposed is the magnetic property. Designing magnetic scaffolds has been proposed for the regeneration and repair of tissues in damage and disease [10]–[15]. The processing strategy in these studies primarily involved dip-coating of the scaffolds in aqueous ferro-fluids containing iron oxide nanoparticles (NPs). Importantly, scaffolds coated with magnetic NPs (MNPs) more avidly attract growth factors and other biomolecules *in vitro* [10]–[15]. Moreover, the incorporation of MNPs into scaffolds is believed to increase the rate of bone cell growth and differentiation due to the bone tissue ability to recognize the mechano-electrical conversion (i.e., mechanical stress converted from or into a magnetism-induced voltage) leading to an increased cellular proliferation and expression levels of multiple genes related with bone differentiation [16], [17].

Due to their biocompatible and nontoxic characteristics, MNPs, mainly superparamagnetic iron oxide NPs, are being used in many biomedical applications, such as magnetic resonance imaging, drug delivery, cell and tissue targeting, and hyperthermia [18], [19]. Especially for hyperthermia therapy, the design of nanometric heat-generating sources, which can be activated remotely by the application of an external alternating magnetic field, is desirable. This noninvasive technique could provide local therapy and controlled drug release that induces the growth and differentiation of specific tissues [20]. However, the clinical applications of the magnetic field for targeted therapy have some limitations. In this sense, a proper design of the MNPs into formulations of implantable biomaterials or tissue engineering scaffolds constitutes one of the most attractive research areas in repair and regeneration of tissues including bone [21], [22]. Scaffolds will act as a guiding and stimulating matrix for the cells, providing magnetic activity to the personal needs of the patient.

Among the scaffold forms, nanofibrous matrices resemble the natural fibrillar structure of extracellular matrices, supporting necessary functions for cellular growth and possible specified differentiation [23], [24]. Degradable biopolymer nanofibers produced by electrospinning have been the most widely studied. Desirable characteristics of these biopolymer nanofiber scaffolds include nanotopology, biodegradability, high porosity, and a high surface area to volume ratio, which facilitates cellular anchorage, migration and proliferation, as well as stimulating differentiation associated with nanotopological cues [25].

With this in mind, here we developed a novel biopolymer nanofiber scaffold with magnetic property by means of incorporating superparamagnetic iron oxide (‘magnetite’) MNPs. The processing tools to generate the magnetic nanofibers are described, and the physico-chemical and mechanical properties were investigated. For specific targeting hard tissues, the magnetic nanofibers were cultured with mesenchymal stem cells (MSCs) to assess *in vit*ro proliferation, penetration, and osteogenic potentials. *In vivo* tissue responses were observed by implantation of magnetic nanofiber scaffolds in subcutaneous sites as well as in segmental defects in rats. The series of results on the physico-chemical, mechanical and biological properties are considered to provide useful information on the possible usefulness and potential of the magnetic scaffolds for bone regeneration purpose.

## Experimental part

### Preparation of magnetite NPs

Ferrous chloride tetrahydrate (FeCl_2_⋅4H_2_O) in 1 M HCl and ferric chloride hexahydratate (FeCl_3_⋅6H_2_O) were mixed at room temperature (Fe^2+^/Fe^3+^ = ½). The mixture was dropped into 200 ml of 1.5 M NaOH solution with vigorous stirring for about 30 min. The resulting precipitate was isolated using a magnetic field and the solution was decanted by centrifugation at 8000 rpm. The separation procedure was repeated twice and 200 ml of 0.02 M HCl solution was added to the precipitate with continuous agitation. The product was separated by centrifugation (8000 rpm) and dried at 40°C. All steps were performed under an atmosphere of nitrogen gas. The magnetite NPs were dispersed in citric acid solution (0.05 M) under magnetic stirring, and the pH was adjusted to 5.5 by NH_3_ solution. After 4 h, the magnetite NPs were precipitated by addition of acetone, washed with acetone by magnetic decantation to remove the redundant citric acid, and then dried at 40°C. The surface of the magnetic NPs was coated with citric acid via the constituent COOH groups.

### Electrospinning of MNP-PCL into nanofibers

PCL (MW = 80,000; Sigma-Aldrich) solution was prepared by dissolving 10% w/v PCL in dichloromethane (DCM) and ethanol at a DCM:ethanol ratio of 4∶1. The prepared MNPs dispersed in DCM:ethanol were made into composite solutions with PCL solution. The concentrations of MNPs in PCL were determined at 5, 10, 15, and 20 wt%. The solutions were ultrasonicated to prepare homogeneous and stable nanocomposite solutions. This mixture was loaded into a 10 ml plastic syringe equipped with a 21-gauge needle made of stainless steel. The needle was connected to a high-voltage power supply. The voltage for electrospinning was 15 kV. The tip-to-collector distance was kept at 10 cm and the injection rate was 0.5 ml/h. During the electrospinning process, the drum was rotated at a constant speed by a DC motor to collect the developing nanofibers. All experiments were performed at room temperature.

### Characterizations

The crystal structure was determined by X-ray diffraction (XRD; Ragaku). The samples were scanned in the range of diffraction angle 2θ = 10–60° at a rate of 2° min^−1^ with a step width of 0.02°, 2θ using Cu Kα1 radiation at 40 kV and 40 mA current strength (Rigaku). Thermogravimetric analysis (TGA) was carried out to determine the thermal behavior and compositional fraction of the nanocomposite scaffolds. Fourier transformed infrared (FT-IR, Perkin-Elmer) spectroscopy was used to observe the chemical status of the nanofiber scaffolds. The surface electrical properties of the samples were observed by means of ζ-potential measurement (Zetasizer Nano; Malvern Instruments) at a pH of 7.0 at 25°C. The morphology of the samples was characterized by scanning electron microscopy (SEM) using a model S-3000H microscope (Hitachi), and the internal structure and existence of the MNPs was investigated by transmission electron microscope (TEM) using a model 7100 microscope (JEOL).

The water affinity of the nanofibers was observed by measuring the water contact angle using a Phoenix300 analyzer. The water droplet images made onto the nanofiber samples were observed using a viewing system until the equilibrium shape was achieved at 25°C. The equilibrium status of the droplet water was observed to be attained in a similar fashion to that of the flat cover slip, and a typical image at an equilibrium state was taken for each sample. Data were recorded for up to 1 min and five samples were tested for each group.

### Tensile mechanical tests

The mechanical properties of the nanofiber scaffolds with various compositions were evaluated using an Instron 3344 universal testing instrument at a cross-head speed of 10 mm/min. Membranes were prepared with a thickness of approximately 150–200 µm and then cut to a size of 30 mm×4 mm (gauge length 10 mm), after which tensile load was applied [26]. Stress-strain curves were recorded from the test. The thickness of each membrane was determined from the average value observed on SEM images for five samples for each group. Mechanical properties including tensile strength, yield strength, elastic modulus, elongation, and strain at yield were calculated from the stress-strain curves. Four specimens were tested for each composition.

### Measurement of magnetic properties

The magnetic properties of the samples were studied by a superconducting quantum interference device (SQUID; Quantum Design MPMS-XL7) in an applied magnetic field of ±20 kOe at room temperature [27]. The SQUID was calibrated using a standard reference (high purity nickel sphere), supplied with the instrument. Magnetic properties of the scaffolds were evaluated in terms of saturation magnetization and hysteresis loop area.

### Apatite forming ability and degradation tests *in vitro*

Each PCL specimen used in this experiment was immersed in 200 ml of aqueous 2M NaOH with vigorous stirring at 30°C for 4 h. The specimen was removed from the NaOH solution, washed extensively with ultra-pure water, and dried at room temperature in air for a few minutes. The soaking regimen was designed to activate the hydrophobic surface by the creation of carboxyl groups that then allow the bonding of calcium and phosphate ions [28]. The NaOH-treated nanofibers were alternately dipped in calcium ion and phosphate ion solutions by the following process (abbreviated as CaP treatment). The NaOH-treated nanofiber was dipped in 100 ml of 150 mM CaCl_2_ aqueous solution for 10 s, dipped in 20 ml of ultra-pure water for 10 s, and then dried in air for a few minutes. The specimen was subsequently dipped in 100 ml of 200 mM Na_2_HPO_4_ aqueous solution for 10 s, dipped again in 20 ml of ultra-pure water for 10 s, and then dried in air for a few minutes. The alternate dipping was performed three times at room temperature. The same CaCl_2_ and Na_2_HPO_4_ solutions, and ultra-pure water, were used for a given specimen throughout the three cycles of alternate dipping. The NaOH-treated specimens and those further subjected to the CaP treatment were immersed in 45 ml of (1.5×) simulated body fluid (SBF) of pH 7.4 and ion concentrations (Na^+^ 142.0, K^+^ 5.0, Mg^2+^ 1.5, Ca^2+^ 2.5, Cl^−^ 147.8, HCO_3_^−^ 4.2, HPO_4_^2−^ 1.0, SO_4_^2−^ 0.5 mM), approximating those present in human blood plasma, at 37°C for various days for the growth of nanocrystalline bone mineral. The specimen, after removal from the fluid, was gently washed with ultra-pure water.

The hydrolytic degradation property of the magnetic nanofiber scaffolds was examined by immersing the samples in a saline solution (phosphate buffered) at 37°C for up to 28 days. The weight change (weight loss) of the samples was recorded during the test period. SEM images of the samples before and after the degradation test were also taken.

### Isolation and culture of rat bone marrow MSCs

MSCs derived from rat bone marrow were isolated as described previously [29]. All protocols involving animals were approved by the Animal Care and Use Committee of Dankook University. In brief, the proximal and distal epiphyses femora and tibiae of male adult Sprague-Dawley rats (180–200 g) were cut off and bone marrow tissue was flushed out with dispase II and type II collagenase solution in α-Minimal Essential Medium (α-MEM). The tissue was centrifuged and resuspended in normal culture medium (α-MEM supplemented with 10% fetal bovine serum containing 1% penicillin/streptomycin) and then placed in a culture dish in an incubator under a humidified atmosphere of 5% CO_2_ in air at 37°C. Nonadherent hematopoietic cells were removed from the medium during medium changes done every 3 days, and the cells that had undergone three passages were used for further experiments. Experiments were performed in an osteogenic medium, containing 50 µg/ml ascorbic acid, 10 mM β-glycerol phosphate and 10 nM dexamethasone.

### Cell adhesion assays

Nanofibrous scaffolds with different compositions were sterilized in ethylene oxide. Samples were prepared with sizes fit to the wells of 24-well plates and then fixed with plastic ring to prevent floating. MSCs were seeded at a density of 5×10^3^ onto each sample. At different culture times (2, 4, 8, and 16 h) to allow initial adhesion steps, the cells adhered on the nanofibrous samples were assessed by Trypan blue screening and hemocytometer counting. Five replicates were tested for each experimental condition. The adhesion and spreading shapes of cells were visualized by confocal laser scanning microscopy (CLSM). Cells cultured at different periods were immunostained by fixation with 4% paraformaldehyde for 5 min, permeation with 0.3% Triton X-100, blocking with 2% bovine serum albumin, and incubated with primary antibodies against FAK (A-17) (1∶100; Santa Cruz Biotechnology) at 4°C overnight. Cells were double-labeled with the appropriate fluorescein isothiocyanate (FITC)-conjugated secondary antibody in combination with Alexa Fluor 546-conjugated phalloidin diluted in phosphate buffered saline (Invitrogen) for 1 h to stain F-actin. The nuclei were stained with 4′,6-diamidino-2-phenylindole (DAPI) and the images were captured using a LSM700 confocal microscope (Carl Zeiss).

### Cell penetration assays

To assess the cell penetration into nanofibrous scaffolds, the *z*-stack images of cells were obtained using LSM700 META (Carl Zeiss) in the range 0–75 µm (the approximate thickness of nanofiber scaffolds) with 1 µm interval under a 3-color DIC configuration mode (488 nm for FITC, 555 nm for Rode, and 420 nm for DAPI and DIC). The *z*-stack images were converted into three-dimensional (3D) projection images by the orthogonal tool in the ZEN software. Furthermore, the side-view of *z*-stack images was visualized by the *z*-stack series side view tool. Based on the side-view images, the cell localization was quantified to represent average penetration depth.

### Alkaline phosphatase (ALP) determination

The osteoblastic differentiation of MSCs cultured on the magnetic nanofiber scaffolds was first assessed by determining ALP activity, which is a relatively early osteogenic marker. After culture for 7 and 14 days, cells were gathered from the nanofiber scaffolds and disrupted with lysis buffer by cycles of freezing and thawing. Samples were added to the ALP reaction medium for enzymatic reaction according to the manufacturer's instructions (Sigma-Aldrich). The sample quantity added was determined based on total protein content, when measured with a commercial DC protein assay kit (BioRad). Generation of *p*-nitrophenol was measured spectrophotometrically at an absorbance of 405 nm. Five replicate samples were used for each experimental condition.

### Osteoblastic gene expressions by quantitative reverse transcription-polymerase chain reaction (RT-PCR)

The expression of mRNA levels of the bone-associated genes, including collagen type I (Col I), osteopontin (OPN), and bone sialoprotein (BSP), were determined quantitatively by real-time PCR (*Q*PCR). After culture for 7 or 14 days, cells were gathered from each sample and total RNA was isolated using the RNeasy Mini Kit (Qiagen), according to the manufacturer's instructions. The RNA was reverse-transcribed to cDNA using the Superscript kit (Invitrogen) with random hexamers as primers. PCR amplification was performed using Sensimix Plus SYBR Master Mix (Quantace). A comparative CT method was used for analysis by normalizing the PCR product accumulated for each gene to the beta-actin housekeeping gene.

### *In vivo* compatibility in rat subcutaneous tissue

Electrospun nanofibrous scaffolds (PCL, 5MNP, 10MNP and 15MNP) were prepared with a dimension of approximately 1.5 cm×1.5 cm×300 µm, and then sterilized with ethylene oxide before surgical operation. For the tissue compatibility test, male Sprague-Dawley rats were used. The animal experiments were approved by Dankook University Institutional Animal Care and Use Committee. All manipulations on animals were carried out under general anesthesia with sterile conditions. Rats were divided into four groups (one animal per group). Each rat received an intramuscular injection with ketamine 80 mg/kg body weight and xylazine 10 mg/kg body weight. Four small subcutaneous pouches were created with scissors in the back area laterally from the spine in each animal and each scaffold specimen was placed in the defect. The incision was sutured with 4-0 non-absorbable monofilament suture (Prolene) and the rats were observed for 4 weeks under an alternating 12 h light/12 h dark schedule. Standard pellet food and water were provided *ad libitum*.

Animals were sacrificed at 4 weeks. The implantation zone with adjacent tissues was harvested for histologic analysis and immediately immersed in 10% neutralized buffered formalin for 24 h at room temperature, dehydrated in a graded ethanol series, bisected, and embedded in paraffin. Histological samples 5 µm in thickness were prepared using a rotary microtome, and were stained with hematoxylin and eosin (H&E) or Masson's trichrome (MT) using established regimens. Sample slides were observed with an optical microscope for tissue compatibility and blood vessel formation. Histological scoring (0–4; 0 being lowest and 4 being highest) was made based on different histopathological features including immune response, fibrous capsule thickness, presence of blood vessels, and proliferation of fibroblasts.

### *In vivo* bone formation ability in rat radius segmental defect

After confirming the tissue compatibility in subcutaneous tissue, the bone forming ability of the magnetic nanofiber scaffolds was investigated using a rat segmental bone defect model. Six Sprague-Dawley rats were used in this study. The animal care and housing protocols were same as the tests described in a previous section.

The hairs of the forelimbs were shaved and the region was aseptically prepared using povidone and 70% ethanol for surgery. Following aseptic procedures, a 15-cm skin incision was made along the lateral side of the front right or left limb, superficial to the radius bone. The muscles were blunt dissected and the periosteum was elevated to obtain sufficient surgical field for radial diaphysis resection. Two segmental bone defects with lengths of 5 mm were prepared in each rat from the center section of the radius bone using a burr with copious irrigation with sterile, cooled saline to prevent damaging the bone and to wash out bone particles. Animals were randomized into three groups. Each defect were randomly implanted with two types of nanofiber scaffolds (PCL and 15MNP) or kept empty as a negative control. In case of nanofibrous scaffolds, each sample was rolled around the defect and the remaining part of radius bone to completely cover the region and to prevent soft tissue invasion into the defect. The fascia was closed with 4-0 absorbable materials and skin incisions were sutured with 4-0 non-absorbable materials. The animals were allowed to function immediately on the operated limbs.

The animals were monitored for 8 weeks postoperatively. After sacrifice, the skin was dissected and the samples and its surrounding tissues were harvested along with surrounding bone using a motor drill equipped with a diamond wheel. The specimens were fixed in 10% buffered neutralized formalin for 24 h at room temperature and prepared for histology. Fixed samples were demineralized, dehydrated, and subsequently embedded in paraffin. Five micrometer serial longitudinal sections were prepared and then treated by the H&E and MT staining methods and examined using a light microscope.

### Statistical analysis

Data are presented as mean ± one standard deviation. Statistical analysis was performed using one-way analysis of variance (ANOVA) followed by Tukey's test, comparing independent sample groups. Significance was considered at p<0.05 or p<0.01.

## Results

### MNPs and magnetic nanofibers

**Figure 1:**
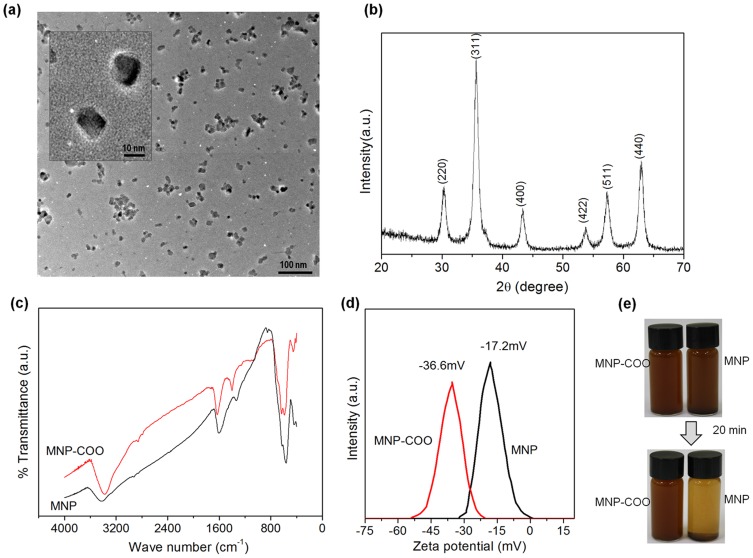
Characteristics of the MNPs prepared with citric acid functionalization. (a) TEM image showing ultrafine monodispersed nanoparticles with an average size of 12.0 nm. (b) XRD pattern with typical magnetite peaks. (c) FT-IR spectrum before and after the citric acid functionalization. (d) Zeta potential of the MNPs before and after the citric acid functionalization, showing more negatively charged with the functionalization. (e) Dispersion ability of the MNPs in distilled water and DCM/ethanol (a solvent used for the nanocomposite electrospinning with PCL), showing the citric acid functionalization increased stability significantly.

MNPs produced by the citric acid functionalization were characterized (**Fig. 1**). TEM of the MNPs revealed monodispersed ultrafine nanoparticles with an average size of 12±1.34 nm (**Fig. 1a**). XRD pattern included peaks at (220), (311), (400), (422), (511), and (440), characteristic of magnetite at around 2θ≈30°, 35°, 43°, 53°, 57°, and 63°, respectively (**Fig. 1b**), identifying a cubic spinel structure according to JCPDS card # 019-0629. The average diameter of MNPs was determined from the XRD pattern according to Scherrer's equation: D = kλ/β cosθ [30], where λ is X-ray wavelength of Cu Kα radiation 1.54 A°, k is the shape factor that can be assigned a value of 0.89 if the shape is unknown, θ is Bragg angle, and β is the full width at half maximum in radians. The most intense peak (311) was chosen to calculate the average diameter, which gave an estimated average size of about 10.8±1.21 nm, similar to the result from TEM image. MNP chemical bond structure before and after the citric acid functionalization was analyzed by FT-IR in the 4000 to 400 cm^−1^ region (**Fig. 1c**). The IR spectrum of MNPs showed bands at 3426 and 1606 cm^−1^, assigned to ν OH and δ OH, respectively. In the citric acid functionalized MNPs, a large and intense band at 3426 cm^−1^ confirmed the presence of water traces, while the absorption at 3200–3400 cm^−1^ suggested the presence of non-dissociated OH groups of the citric acid. Furthermore, a band at 1630 cm^−1^, assigned to the symmetric stretching of OH from COOH group, revealed the binding of a citric acid radical to the magnetite surface. The neighbor band at 1401 cm^−1^ was characteristic of an asymmetric stretching of COOH groups [31]. The strong bands at 600-400 cm^−1^ and 578 cm^−1^ are characteristic of the Fe-O structure of the magnetic core particles [27]. The results confirmed that the citric acid bound chemically to the magnetite surface by carboxylate chemisorptions, resulting in citrate ions. The zeta potential of MNPs before and after the citric acid functionalization, measured at pH 7.0, was indicative of the highly negatively charged characteristic of the nanoparticles (−17.2 mV) and the creation of increased negative charge with the citric acid functionalization (−36.6 mV), suggesting the effect of citrate ions present on the NP surface (**Fig. 1d**). The dispersion ability of the MNPs in distilled water or in organic solvent DCM/ethanol, as was presently used for the nanocomposite electrospinning, is important to determine when evaluating the dispersion of the nanoparticles in the solvent (**Fig. 1e**). The citric acid functionalized MNPs efficiently dispersed in either of the solvents, preserving the stability over hours. This stability was not readily observed in the absence of citric acid functionalization. These observations indicated the effectiveness of the citric acid functionalization in preserving stable solution and consequently enabling nanocomposite solution with PCL.

**Figure 2:**
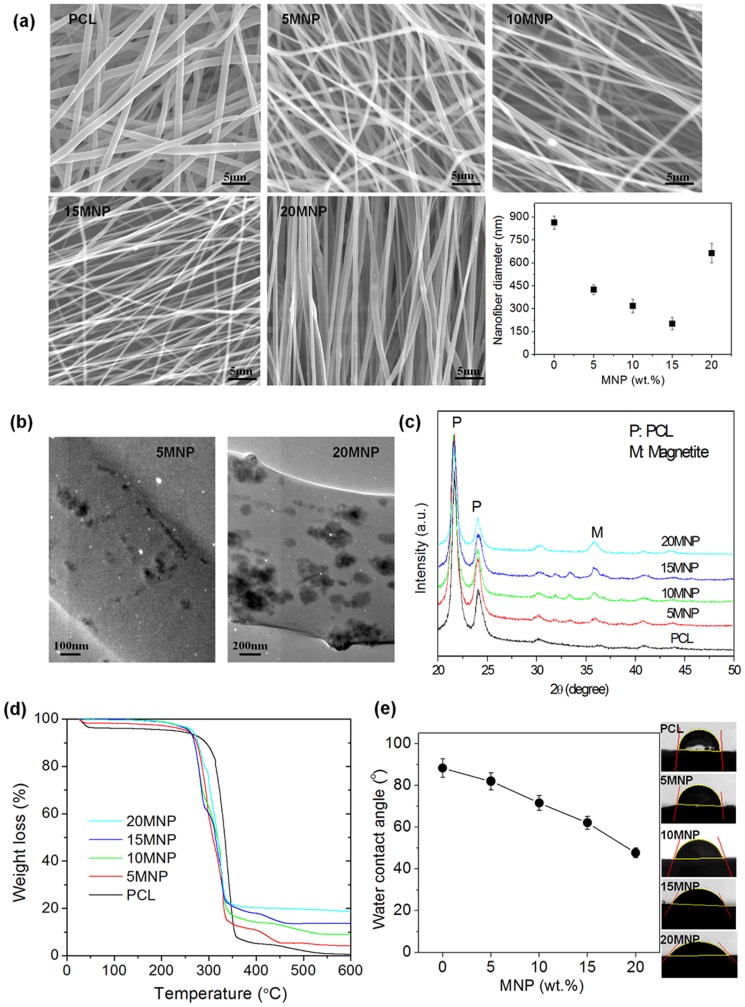
Morphological and behavioral analyses of MNPs. (a) SEM morphology of the electrospun nanofibers with different compositions (PCL or its nanocomposite with MNPs at varying concentrations; 5% ‘5MNP’, 10% ‘10MNP’, 15% ‘15MNP’ and 20% ‘20MNP’) and the nanofiber diameter measurement. (b) TEM image shows the internal structure revealing nanoparticles present in the nanofiber (‘10MNP’ and ‘20MNP’). (c) XRD patterns of the nanocomposite nanofibers. (d) TGA results of the weight loss of samples during thermal treatment. The remained weight was 4.1, 9.0, 13.6 and 18.6%, respectively, for 5MNP, 10MNP, 15MNP and 20MNP, demonstrating slightly lower values than but well matching to the amounts initially added to the nanocomposite solutions. (e) Wettability test of the nanofiber scaffolds using a contact angle tester, showing significant improvement in wettability (contact angle decrease) with incorporation of MNPs.

MNPs dispersed in DCM/ethanol were added with PCL to prepare a nanocomposite solution, which was then followed by an electrospinning to generate nanofibrous scaffolds. Among the possible variables dominating the electrospinning process [7], the solution viscosity that was controlled by changing the polymer concentration was the major factor, which also affected the fiber size and morphology. Electrospinning of nanocomposite colloidal solutions containing MNPs at varying concentrations (5, 10, 15, and 20%) generated membranes consisting of well-defined nanofibers. **Fig. 2a** shows a representative SEM image of the nanofibers, with the typical smooth, uniform, and bead-free morphology. It is clear in this image that smooth and continuous fibers are formed by electrospinning of pure PCL. The measured nanofiber diameters were 864 (±43), 425 (±31), 318 (±46), 202 (±40), and 664 (±63) nm, respectively, for PCL, 5MNP, 10MNP, 15MNP, and 20MNP. While the addition of MNPs up to 15% progressively and gradually decreased the nanofiber diameter, the addition of 20% increased the fiber diameter. It is considered that other factors such as solution electrical conductivity and viscosity should be changed with the addition of MNPs, which affected the fiber diameter significantly. While the presence of MNPs could not be revealed by SEM, MNP addition made the nanofiber scaffold more dark brown in color.

The internal structure of the nanocomposite nanofibers was observed by TEM (**Fig. 2b**). Sampling was achieved by placing the TEM grid very close to the tip opening of the syringe needle for a few seconds during the electrospinning process. Characteristic images of 10MNP and 20MNP are shown. NPs within the PCL polymer matrix were readily apparent. NPs were distributed in 5MNP with individual particles being relatively well-separated, while those in 20MNP were agglomerations of several dozen NPs evident.

XRD patterns of the nanofibers showed typical peaks related with PCL (‘P’) and MNPs (‘M’) (**Fig. 2c**). Thermogravimetric analysis of the samples provided information on the quantity of MNPs present in the samples. Similar thermal behaviors were observed for all samples (**Fig. 2d**). The slight weight loss below 250°C was attributed to water removal, while the second weight loss at approximately 360°C was attributed to the decomposition of PCL polymer. Decomposition finalized at approximately 600°C. The remnant weight was 4.1, 9.0, 13.6, and 18.6%, respectively, for 5MNP, 10MNP, 15MNP, and 20MNP, demonstrating slightly lower values, but nonetheless well-matched, to the amounts initially added to the nanocomposite solutions. The wettability of the nanofibers was investigated with contact angle testing (**Fig. 2e**). While the PCL nanofibers displayed a large contact angle of approximately 88°, the nanocomposite nanofibers displayed gradually decreased contact angle values (down to approximately 68° with 10% MNPs and about 47° with 20% MNPs), suggesting the improvement of wetting property (water affinity) of the MNP-added nanofiber scaffolds. This improvement was due to the presence of carboxylated MNPs.

### Tensile mechanical properties

**Figure 3:**
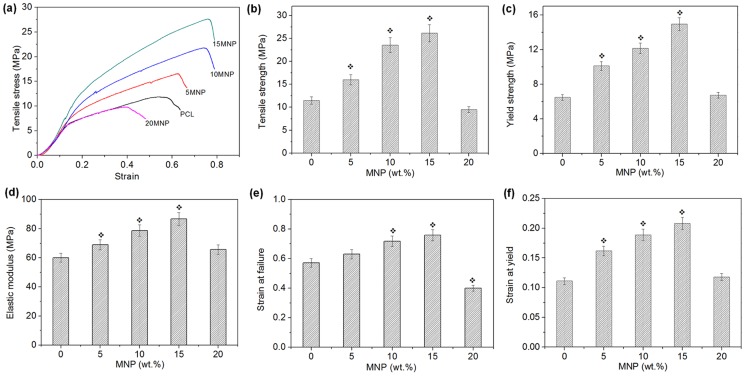
Tensile mechanical properties of the PCL and PCL-MNP nanocomposite nanofiber scaffolds. (a) stress-strain curve, (b) tensile strength taken from the maximum stress prior to a failure, (c) yield strength, measured at yield point, (d) elastic modulus, taken from the initial slope in stress-strain curve, (e) strain at failure, taking as elongation rate, (f) strain at yield, measured at yield point. The addition of MNP up to 15% gradually increased all the mechanical properties measured, which however, decreased with further addition of 20%. Tests were carried out on five individual specimens and average and one standard deviation values were plotted.

The mechanical properties of the nanocomposite scaffolds were measured by tensile strength testing. **Fig. 3a** shows the typical tensile stress-strain curves for the samples (PCL and PCL-MNP nanocomposite nanofibers). All samples showed a similar stress-strain behavior with two discernible stages: an initial stage having a rapid increase in stress up to a strain of approximately 0.1–0.15, followed by a stage featuring a slowing of the stress increase up to the failure point. Based on this stress-strain curve, important mechanical parameters including tensile strength, yield strength, elastic modulus, strain at failure, and strain at yield were analyzed. Tensile strength, measured as the maximum strength prior to failure, increased as the MNP content increased up to 15%; 11.5 MPa in pure PCL became 26.2 MPa in 15MNP (**Fig. 3b**). However, the addition of 20% MNP reduced tensile strength down to 9.5 MPa. The yield strength measured at the yield point also behaved in a similar way (**Fig. 3c**); 6.5 MPa in pure PCL became 15.0 MPa in 15MNP and then 6.7 MPa in 20MNP. The elastic modulus of the nanofibers (calculated within 0.5% of strain range), representing stiffness of samples, was measured by the initial slope of the stress-strain curves (**Fig. 3d**). There was also a marked increase with the MNP addition from 60.1 MPa in pure PCL to 86.7 MPa in 15MNP, which again was reduced to 65.6 MPa in 20MNP. Along with the strength and stiffness values, the elongation behaviors were also assessed. The strain at failure, considered as the elongation rate, increased slightly with the MNP addition up to 15% (from 0.57 to 0.75), which however was reduced markedly to 0.4 with 20% addition (**Fig. 3e**). The strain at yield point increased significantly with the MNP addition up to 15% (from 0.11 to 0.21), which was again reduced (0.12) with 20% addition.

### Magnetic properties

**Figure 4:**
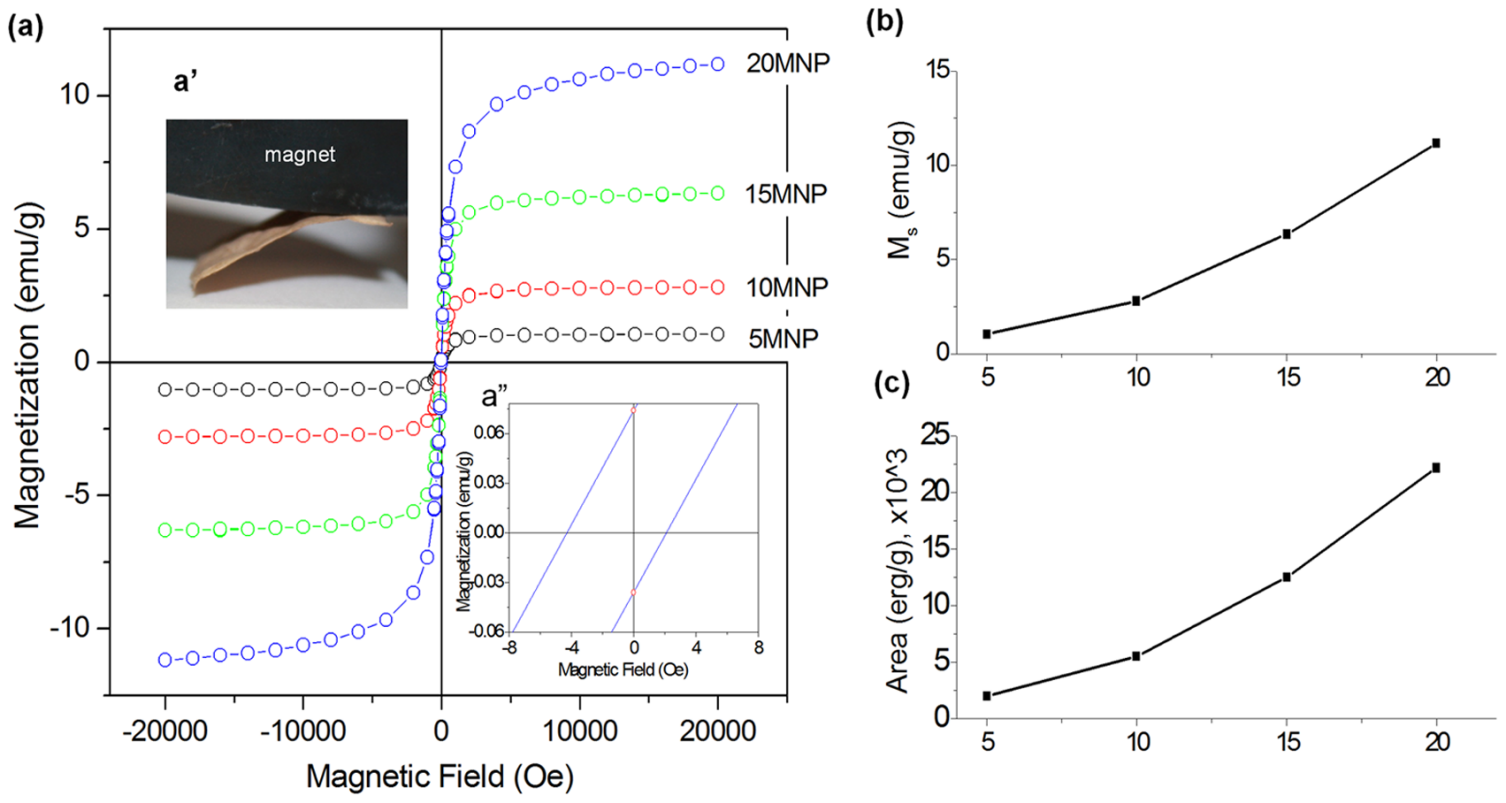
Magnetic properties of the nanocomposite nanofiber scaffolds. (a) Magnetization curve as a function of an applied magnetic field, measured with SQUID magnetometry, showing a hysteresis loop. Inset (a′) shows optical view of nanofiber samples attracted to a magnet. Inset (a″) shows magnetization behavior at low magnetic field, revealing coercivity (*H_c_*) and remnant magnetization value (*M_r_*). The magnetic behavior was typical of weak ferromagnetic or superparamagnetic materials. (b) Saturation magnetization, *M_s_*, and (c) integrated loop area, measured from the magnetization curves. Increasing MNP content increased the *M_s_* and loop area.

The magnetic properties of the nanocomposite nanofiber scaffolds were assessed by the room temperature hysteresis loop using a SQUID magnetometer (**Fig. 4**). The magnetization curve indicates the magnetization as a function of an applied magnetic field. All samples showed a typical hysteresis loop with the magnetic field change from +20 to −20 kOe (**Fig. 4a**). It was readily evident that the nanofiber samples showed a strong attraction to a magnet (**Fig. 4a**, inset a′). The magnetization behavior at low magnetic field range (−8 Oe to +8 Oe) is also presented (**Fig. 4a**, inset a″). From this, the scaffolds showed a moderate coercivity (H_c_) of 2.5 Oe, and a low remnant magnetization (M_r_) of 0.27 emu g^−1^, considered as weak ferromagnetic or superparamagnetic materials as these have typically narrow hysteresis loop and a low coercivity [32]. The saturation magnetization ranged 1.0–11.2 emu/g with increasing value as the content of MNP increased (**Fig. 4b**), and was related to the relative mass fraction of the MNPs incorporated in the PCL polymer matrix. Another important parameter is the magnetic loss/cycle or the area of the hysteresis loop. The integrated loop area was calculated for a maximum applied field of ±20 kOe. The value increased as the MNP content increased, similar with the behavior of saturation magnetization (**Fig. 4c**).

### *In vitro* apatite forming ability and hydrolytic degradation

**Figure 5:**
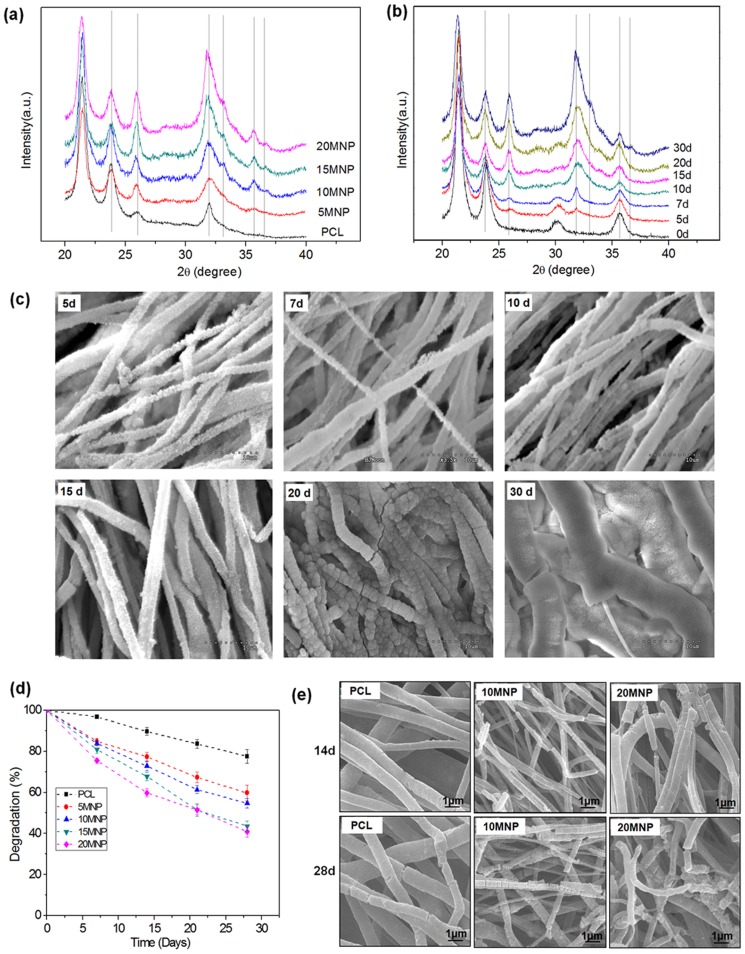
Degradation analyses of MNPs. (a–c) Apatite forming ability in 1.5SBF and (d,e) degradation behavior of the PCL-MNP nanofiber scaffolds; (a) XRD pattern of different samples taken at 30 days of 1.5SBF-immersion. (b) XRD pattern of 20MNP at varying immersion times. (c) SEM morphology showing the formation of apatite crystal on the surface of nanofiber (20 MNP). (d) Weight change due to a degradation in PBS at 37°C for periods up to 28 days. (e) SEM morphology of the nanofiber samples after the degradation test for 14 and 28 days.

As PCL-MNP nanocomposite nanofibers have been considered for use as bone regenerative matrices, we investigated the apatite forming ability *in vitro* in simulated body fluid. Here we used 1.5-times concentrated SBF (1.5SBF) to accelerate apatite formation and shorten the investigation period. Each sample was immersed in 1.5SBF for different periods (0, 5, 7, 10, 15, 20, and 30 days) and then removed for analyses of the changes in phase and morphology. After 30 days of immersion, the XRD pattern of all samples was observed (**Fig. 5a**). In pure PCL, apatite peaks at 2θ values of ∼26° and ∼32°, assigned to (002) and (211) reflections of HA crystallites (JCPDS card #. 74-0565), developed weakly. As the MNP content increased, the peak intensities increased. Some other apatite peaks also developed while several PCL peaks disappeared. These results demonstrated the enhanced apatite forming ability of the nanofiber scaffolds with the addition of MNPs. Apatite formation occurred gradually with time, as observed in 20MNP samples as a function of immersion period (**Fig. 5b**). SEM morphology of samples after the SBF test was observed (**Fig. 5c**). Some tiny nanocrystallites that formed initially (5 days) became bigger with time. The apatite crystalline phase covered the nanofiber surface almost completely at the intermediate period and filled the nanofiber interspaces at a longer period (>20 days).

Along with the SBF tests, degradation behaviors of the nanofiber scaffolds were investigated in PBS at 37°C. Weight change was recorded during the test for a period up to 28 days (**Fig. 5d**). Weight decreased linearly with the time of culture for all nanofibrous scaffolds. The weight loss was more substantial in the nanocomposite nanofibers as the MNP content increased. After 28 days, the weight loss was approximately 20% for PCL, approximately 45% for 10MNP, and approximately 60% for 20%MNP. The degraded nanofiber samples were observed by SEM; compared to PCL, substantial disintegration was apparent in nanofibrous structure of 10MNP and 20MNP (**Fig. 5e**).

### Adhesion, spreading, and penetration of MSCs

**Figure 6:**
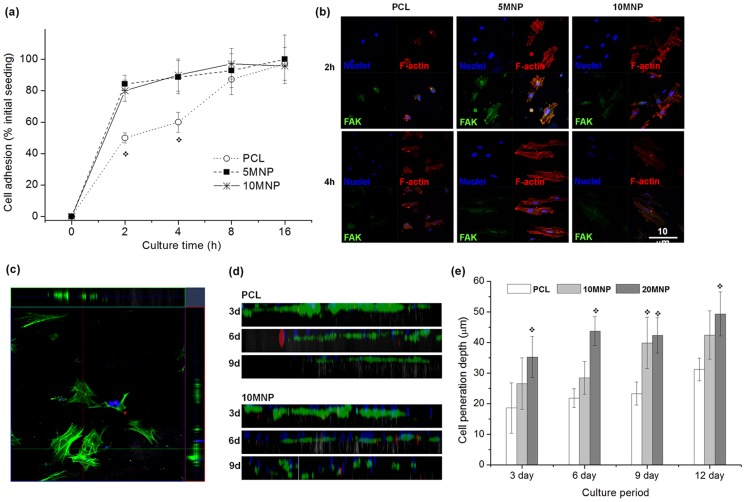
MC3T3-E1 cell adhesion and penetration tests on the nanofiber scaffolds. (a) Initial cell adhesion level on the nanofiber scaffolds during culture for up to 16 h, presented as % initial seeding. Significantly higher levels of cell adhesion noticed on the PCL-MNP scaffolds vs PCL (*p<0.05). (b) Cell adhesion morphology taken from confocal microscopy of immunofluorescent stained cells at 2 h and 4 h of culture; nuclei in blue, F-actin in red and FAK in green. (c–e) Cell penetration assay through the nanofiber scaffolds; exemplar image showing that *z*-stacking of immunofluorescence-stained cells (F-actin in green and nuclei in blue) were unfolded on *xz*- and *yz*-planes to reveal 2D constructed images (c), which were then combined to complete construction of depth profile of cells on 2D plane view (d, compared images of PCL and 10MNP samples at a culture period of 3, 6 or 9 days), and the quantification of depth profile (e, shown average positions of cell penetration depth), showing significant improvement in cell penetration within the nanofibers incorporating MNPs.

The initial adhesion behaviors of MSCs were assessed by enumerating the adherent cells on the nanofibrous scaffolds during culture for 2, 4, 8 and 16 h (**Fig. 6a**). At 2 h, cells substantially adhered to the 5MNP ad 10MNP samples (over 80%), while about 50% of the cells adhered to PCL samples. While the adhesion level in 5MNP and 10MNP reached a plateau around 90–100% thereafter, adhesion in PCL abruptly increased up to 8 h, reaching a level similar to those in magnetic nanofibers. The adhesion and spreading behaviors of MSCs were visualized by CLSM (**Fig. 6b**). Focal adhesion kinase (FAK) was co-stained with F-actin to reveal cytoskeletal processes and focal adhesions of cells. For PCL samples, cells showed little spreading at 2 h, but more spreading at 4 h. For 5MNPs and 10 MNPs, as early as 2 h, cells showed substantial spreading with active cytoskeletal processes and focal adhesion contacts, which were also evident at 4 h. The initial adhesion and spreading behaviors of MSCs were thus confirmed to be significantly improved in the magnetic nanofibrous scaffolds.

Cellular penetration into the nanofibrous scaffolds was investigated during culture for up to 12 days. Cells were stained for F-actin as an indicator of cytoskeletal processes and with DAPI for nuclei to visualize cellular migration within the nanofibrous networks. Confocal images were 3D-constructed to profile green fluorescence signals at 2D areas (**Fig. 6c**, which shows a representative example). The 2D profiled images obtained for each sample are shown in **Fig. 6d**. While the green signals were mostly present at the top side of the field for the case of PCL (even prolonged cultures), the signals diffused to the middle and bottom side of the field for magnetic nanofibers. The average level of green signals was quantified (**Fig. 6e**), which revealed that the cell penetration depth was significantly enhanced in the magnetic nanofiber scaffolds and more so with higher content of MNPs (PCL<10MNP<20MNP).

### Osteogenic differentiation

**Figure 7:**
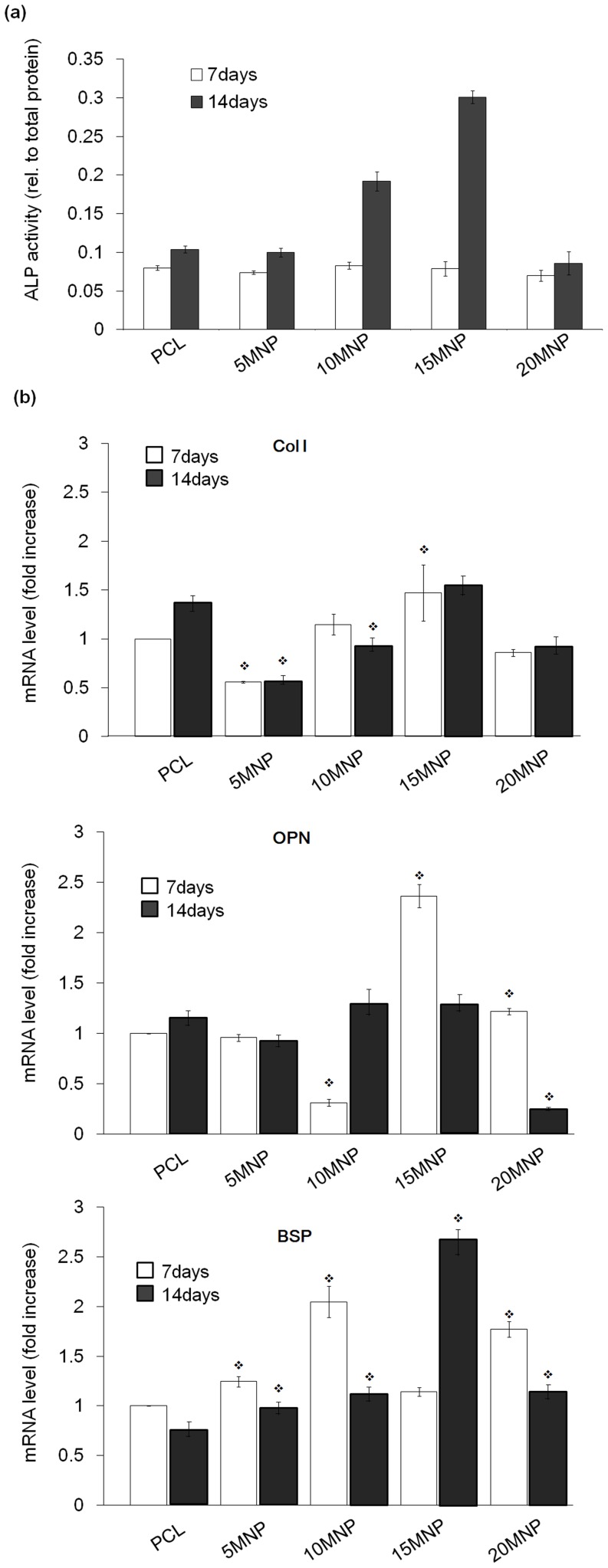
Osteogenic differentiation of cells during culturing on the nanofiber scaffolds for 7 and 14 days. Differentiation was assessed by (a) ALP activity and (b) quantitative RT-PCR, where the mRNA expression of bone-associated genes (Col I, OPN and BSP) was compared between groups.

The osteoblastic differentiation of MSCs cultured on PCL-MNP nanofibrous scaffolds was first assessed by ALP activity (**Fig. 7a**). While there was little difference between samples at day 7, significant improvement was evident at day 14, particularly in the 10MNP and 15MNP scaffolds. Gene expressions of the cells were analyzed by *Q*PCR. The mRNA levels of Col I, OPN, and BSP expressed by the cells were compared (**Fig. 7b**). Gene expression levels were markedly up-regulated, particularly those for OPN and BSP; 2–3 fold increases were evident for OPN in 15 MNP at day 7, and for BSP in 10MNP and 20MNP at day 7 and 15MNP at day 14.

### *In vivo* tissue compatibility and bone forming ability

**Figure 8:**
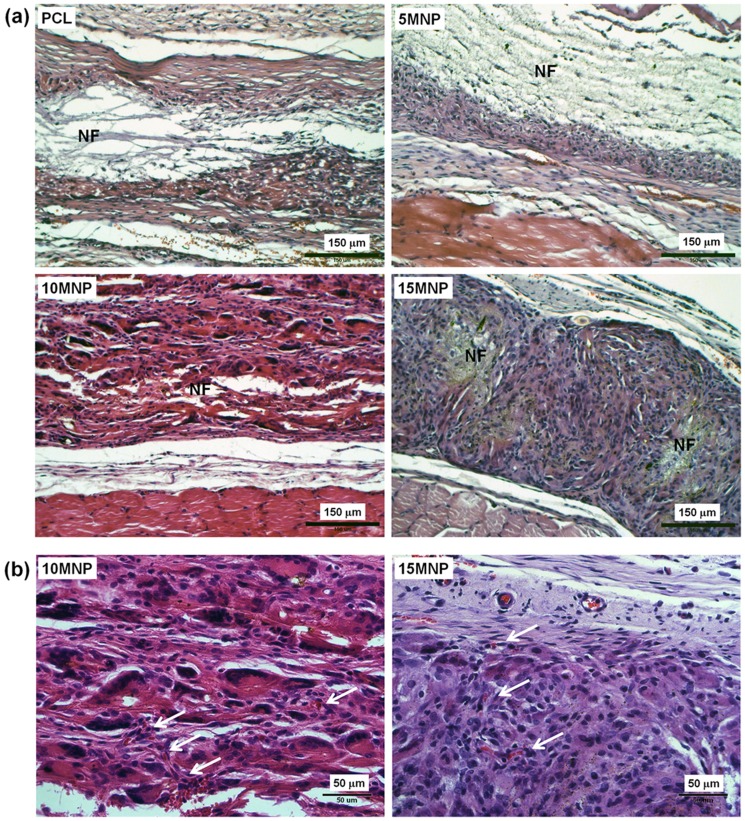
Histological views of the nanofiber scaffolds (PCL, 5MNP, 10MNP and 15MNP) after implantation in rat subcutaneous tissue for 4 weeks. Images at (a) low and (b) high magnification. Tissue samples were H&E stained and visualized under optical microscopy. Connective tissue was formed in areas between the scaffold and adjacent tissues. Neovascularization was also found within the scaffolds (indicated as arrows in (b)). Considerable degradation of scaffolds was noticed particularly in 10MNP and 15MNP, which was not readily observed in pure PCL and 5MNP (‘NF’ in (a) indicates the remaining part of nanofiber scaffold without being degraded). Fibroblasts actively migrated to the site of degradation, and activated fibroblasts were easily detected within the remaining scaffold area.

To assess the tissue compatibility of the PCL-MNP nanofiber scaffolds, samples were implanted in rat subcutaneous tissue for 4 weeks. After surgery, no rats presented inflammatory processes or apparent contamination on their implantation area. Histological views of the sample groups (PCL, 5MNP, 10MNP, and 15 MNP) implanted for 4 weeks are shown in **Fig. 8a**. There were no tissue rejections or significant inflammatory reactions in any sample. Connective tissues made of oriented collagen fibers formed in spaces between the sample and adjacent tissues, and proper neovascularization was also found within the scaffolds. While all four sample groups showed good tissue compatibility, some scaffold degradation was apparent, with the degradation area being replaced by the grown connective tissue, mainly in the 10MNP and 15MNP scaffolds, and not to the same degree in PCL or 5MNP groups (indicated as the different areas of ‘NF’). While PCL and 5MNP retained the compact structure after 4 weeks, the structure of 10MNP and 15MNP became rarefied, and the contours of the scaffold in the areas replaced by the connective tissue were not visualized. Fibroblasts actively migrated to the site of degradation, and activated fibroblasts were easily detected within the remaining scaffold area. The degradation rate was ranked in the order: PCL<5MNP<10MNP<15MNP. **Table 1** presents the results of the histological analyses of the quantified parameters. Lower fibrous capsule formation as well as higher blood vessel formation and fibroblast migration were recorded in the MNP-PCL scaffolds (particularly in 10MNP and 15MNP) than in PCL. The magnified images of 10MNP and 15MNP well revealed the signs of blood vessel formation within the scaffolds (indicated as white arrows in **Fig. 8b**).

**Table 1:** *In vivo* tissue response parameters assessed from the histological images of the subcutaneously implanted samples.

	PCL				5MNP				10MNP				15MNP			
	FC	IR	BV	FB	FC	IR	BV	FB	FC	IR	BV	FB	FC	IR	BV	FB
No 1	2	1	1	1	2	1	1	1	1	1	1	2	1	1	1	2
No 2	2	1	1	2	2	2	1	1	1	1	1	2	1	1	2	3
No 3	2	2	1	1	1	1	2	2	1	1	2	3	1	1	2	3
No 4	2	1	1	2	1	1	1	2	1	1	2	2	1	1	1	3

**Figure 9:**
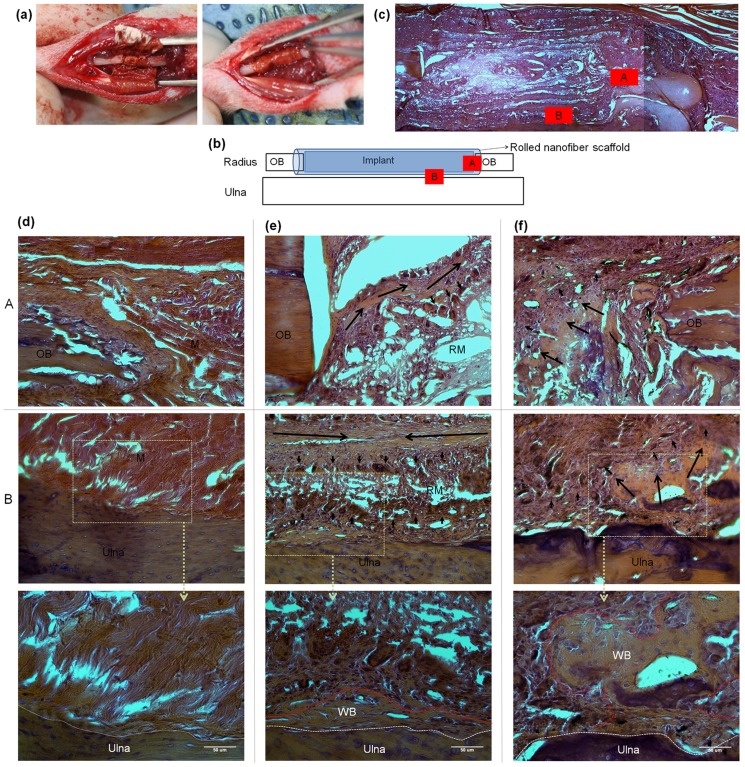
Imaging and histologic analyses of MNPs. (a) Image showing a rat radial segment model; radial segment was removed and then nanofiber scaffold was placed underneath and then rolled to cover the defect region, providing a columnar radial space while preventing soft tissue invasion into the defect region, and the implantation system was schematically drawn in (b), showing relation between outer rolled scaffold, and bone tissue integration within a defect area in radius and the unla in proximity to the scaffold. (c) Representative H&E stained histologic appearance made at 8 weeks after operation. Histologic analysis was made in the regions ‘A’ and ‘B’, and presented in (d) control group free of scaffold, (e) pure PCL scaffolding group, and (f) 15MNP scaffolding group. Enlarged images of bracket regions in ‘B’, showing the new bone formation (woven bone (WB) structure similar to native ulna, and the area indicated as red dotted line). Abbreviations are OB: old bone of radius, M: muscle, RM: remained material, WB: woven bone. Black arrows: bone growing direction, black arrowheads: cell migration from new tissue into material part, white dotted lines: border of ulna and neo-tissue, and red dotted lines: area of WB.

After confirming tissue compatibility of the nanocomposite nanofiber scaffolds, we designed another set of experiment with rat radial segmental model. PCL and 15MNP scaffold groups were representatively tested, and a control group free of scaffold was also tested. **Figs. 9a & b** shows the rat radial segmental model used in this study. The radial segment was removed and the nanofiber scaffold was placed underneath and rolled to cover the defect region, providing a columnar radial space while preventing soft tissue invasion into the defect region. At 8 weeks after operation, H&E stained histological evaluation of bone defects in the nanofiber scaffolding groups (PCL and 15MNP) showed no adverse signs (such as inflammatory reactions) on the surrounding soft tissue and muscle (**Fig. 9c**). However, nonunion was readily observed between the radial osteotomy ends in the scaffold-free control group. The histological views in the regions ‘A’ and ‘B’ were magnified (**Figs. 9d–f**). In the control group, the defects were filled with a thin, loose connective tissue and muscle with minimal new bone formation originating from the edge of the defect margins (**Fig. 9d**, panel A). Muscle contact was apparent on the ulna surface (**Fig. 9d**, panel B). In the PCL scaffold group, new bone formation was noted along the gap (**Fig. 9e**, panel A, indicated by arrows) at the radial osteotomy ends and also on scaffold surfaces. New bone formation started at the surface of the PCL scaffolds and grew with the help of possible scaffold degradation and migration of osteoblasts. As the scaffold covers the remaining parts of the radial bone and prevents fusion of the ulnar and radial bones and soft tissue invasion, new bone formation was frequently observed between the scaffold and the ulna surface (**Fig. 9e**, panel B). In this panel, the arrowheads denote active osteoblasts that are heading for the scaffold at the edge of the newly formed bone. The ulna bone surface exposed to the scaffold side was stimulated, as evidenced by the presence of many osteoblasts that had gathered and migrated to the scaffold. In the 15MNP scaffold group (**Fig. 9f**), bone defect healing similar to the pure PCL scaffold was noted, in terms of bone regeneration occurring at the radial osteotomy ends and on the surface of scaffold. Of special note was that the degree of bone formation was different between the two scaffold groups. In the 15MNP scaffold group, the old bone near defect sites was filled with denser connected tissue and the newly formed bone was better integrated with the edge of host bone than in the PCL scaffold group (**Fig. 9f**, panel A). The 15MNP scaffold showed signs of more rapid degradation than the PCL scaffold; most parts of the scaffold were readily absorbed in conjunction with newly formed bone matrix and osteoblasts that replaced the absorbed areas, and the bone marrow formation was more apparent within regenerated defects than in the PCL group. Remnants of the material were incorporated in the newly formed tissues, and osteoblasts were easily seen (**Fig. 9f**, panel A, arrowheads). The ulna surface also showed a significant degree of cellular response, with active osteoblasts migrating to the scaffold and being impregnated within and the consequent formation of bone matrix in the space (**Fig. 9f**, panel B).

## Discussion

Magnetic scaffolds have recently become the focus of interest mainly due to the possible beneficial effects of magnetic properties on the biological behaviors of cells, such as cell proliferation and tissue-specific differentiation, like hard tissues [21], [22], [33], [34]. Magnetic scaffolds generate a magnetic field to the surroundings, which consequently alter the microenvironment conditions of cells and tissues. Another feature is the generation of heat resulting from the introduced magnetic materials, which enables hyperthermia therapy and magnetism-induced drug delivery and imaging [12], [35], [36]. Therefore, the usefulness of magnetic scaffolds is possibly multi-factorial in terms of the repair and regeneration of damaged and diseased tissues. Despite this potential, the performance of magnetic scaffolds and their mechanism of action have not been adequately studied. Our focus was to produce a nanofibrous form of novel magnetic scaffolds, and address the possible utility for bone regeneration.

The as-prepared MNPs agglomerate easily due to the ultrafine particle size (approximately 12 nm) and weak repulsions between nanoparticles. Therefore, we functionalized the surface with citric acid. The surface functionalization enabled highly negative surface charge potential (−37 mV in ζ-potential), which increased the particle-particle repulsions and homogeneous dispersion in PCL solution (**Figs. 1d,e**). This homogeneous dispersion of NPs free of agglomeration within the solution is the prerequisite for the electrospinning into nanofibers without bead formation [37]. Presently, MNPs added up to 20% could readily generate the nanofiber form without significant bead formation, but the incorporation of MNPs up to 15% was proper in terms of the distribution of NPs within PCL matrix. This was also reflected in the measurement of nanofiber diameter. The addition of MNPs of up to 15% gradually decreased nanofiber size (from 864 nm to 202 nm), while the incorporation of 20% resulted in an abrupt increase (664 nm). When the MNP concentration was low enough to exert repulsions proper for their dispersion in the PCL, the solution properties, such as viscosity and surface tension, as well as the possible magnetic-induced electrical property would give rise to the improvement in electrospinnability and thus decrease in nanofiber size. This was not readily achieved at 20%, at which the viscosity became too high due to agglomerated NPs.

MNPs-added PCL nanofiber scaffolds presented some representative physico-chemical properties. The water contact angle measurements indicated a significant improvement in hydrophilicity with the addition of MNPs, primarily due to the hydrophilic nature of the surface-carboxylated MNPs. This hydrophilic behavior of the nanofiber scaffolds should greatly influence other properties in the biological conditions, such as hydrolytic degradation as well as cell and tissue affinity. Degradation of the nanofiber scaffolds in PBS showed significant enhancement with the addition of MNPs. The hydrophilic nature of MNPs should enable easy and rapid penetration of water molecules into the scaffold matrix, and consequent hydrolysis of the PCL polymer chains. During this process, MNPs are possibly released, giving rise to a total weight loss. Along with the degradation study in PBS, we assessed the apatite forming ability of the nanofiber scaffolds in the medium containing calcium and phosphate ions, with concentrated SBF (1.5×SBF) used to speed up the apatite formation process (and also shorten the test period) [38]. The incorporation of MNPs greatly improved the apatite formation on the surface of the nanofibers, confirming increased bone-bioactivity of the surface. A highly carboxylated MNP surface should help the calcium phosphate deposition, primarily providing nucleation sites, in which calcium ions are attracted first to the negatively-charged surface and subsequently counter-ions like phosphate and carbonate are engaged to form nuclei and precede mineralization [39], [42]. Therefore, the carboxylation of MNPs is considered useful not only for the dispersion of nanoparticles in the solution to enable nanocomposite electrospinning, but also for the apatite mineralization on the surface of nanofiber scaffolds.

Another notable influence of the MNPs was observed in the tensile mechanical properties. Nanofibers containing MNPs at a concentration up to 15% exhibited greater resistance to deformation (based on higher yield point) and final failure (referred from higher tensile strength), as well as enhanced stiffness (higher elastic modulus). However, at 20%MNPs those properties substantially decreased (similar levels to those of pure PCL). In particular, strength improvements were as high as twice that of 15%MNPs. Results clearly demonstrated the strengthening and stiffening effects of the MNPs embedded within the biopolymer nanofibers, and the efficacy was only elicited when the NPs were well-dispersed in the matrix. In this case, the strong chemical interactions between MNPs and PCL polymer chains could ensue. On the other hand, excessive addition of agglomerated nanoparticles could not play a strengthening role. Rather, it could act as a failure origin, resulting in premature deformation and failure [13], [40], [41]. It was notable that those strengthening and stiffening effects of the MNPs were not sacrificed by the reduction in elongation behaviors, not a common phenomenon that can be readily observed in nanocomposites. Rather, the strain values at failure or yield point steadily increased with the incorporation of MNPs. Closer examination of the stress-strain curves revealed that both slopes in the regions up to a yield point and further up to a failure point were substantially higher with MNP incorporation, illustrating the roles in stiffening initially (up to a yield) and then hardening of polymer chain alignment (beyond the yield). These mechanical functions were also facilitated by the possibly strong chemical interactions between MNPs and the polymer chains.

MNP-incorporated PCL nanofiber scaffolds exhibited magnetic behavior over the wide magnetic field range (−20kOe to +20kOe) generally investigated for magnetic materials. The nanofiber scaffolds were readily attracted to a commercial magnet, and showed a hysteresis loop typical of ferromagnetic or superparamagnetic materials based on saturation magnetization and hysteresis loop area. Increase in the content of MNPs gradually increased those magnetic properties; *Ms* of 1–11 emu/g and loop area of 2–22 k erg/g. In general, such magnetic properties are known to change proportional to the volume of magnetic materials. Thus, the volumetric increase of MNPs should add to an increment of magnetic properties. These magnetic behaviors of the nanofiber scaffolds are in fact relatively small compared to those of pure MNPs (*Ms* of 71 emu/g and area of 130 k erg/g) [27]. However, the scaffolds can be seriously considered for the possible applications utilizing the magnetic properties because of their typical characteristics of ferromagnetic hysteresis behaviors and the substantial magnetization level and loop area. Since the area under the loop is proportional to the energy loss and the heat generated by a sample under an alternating field, the nanofibrous scaffolds with a higher concentration of MNPs are capable of generating more heat [27]. Furthermore, the possibility to tune the magnetic behaviors (such as heat generation) dependent on MNP concentration over a wide range provides a means for controllable applications in hyperthermia therapy [42]. This remains to be studied in the near future.

The initial cell behaviors on the magnetic nanofiber scaffolds were significantly improved in terms of cell adhesion, spreading, and migration. Extensive cytoskeleton extensions and expressions of adhesive molecules including FAK were profoundly stimulated with more rapid adherent cell populations on the MNP-incorporated nanofibers. Furthermore, cellular migration through the nanofiber scaffolds, as monitored temporally using immunofluorescence confocal microscopy, was greatly improved in the magnetic nanofiber scaffolds. The incorporation of MNPs enabled cells to better adhere to and spread on the nanofiber scaffolds, and further to propagate through the nanofiber scaffolding channels. These series of cellular events are favored for the utilization of nanofiber scaffolds *in vivo* as tissue regenerative matrices, as well as for their application in *ex vivo* cell culture and tissue engineering constructs. Rapidly attracting surrounding cells and distributing them uniformly through the 3D matrix is the key requirement for the success of implantable and tissue engineering scaffolds. Subsequent responses of cells concerning osteogenesis were also significantly improved with the stimulation of accumulated ALP activity and markedly increased expressions of osteogenic genes, particularly OPN and BSP, which are involved in a later stages of bone differentiation and maturation. The initially stimulated cells in the adhesion and growth stage more rapidly and profoundly switch to later cellular processes involving osteogenic differentiation under proper biochemical cues.

These stimulations in the *in vitro* cellular cultures were further confirmed by *in vivo* rat models. When subcutaneously implanted, the magnetic nanofiber scaffolds preserved the excellent tissue compatibility of PCL-based nanofiber scaffolds with minimal inflammatory tissue reactions. Of particular note was the substantial formation of new blood vessels within the magnetic scaffolds with active cellular migration throughout the scaffolds, while showing considerable degradation signs of the magnetic nanofibrous structure. These findings reflect in part the *in vitro* results on enhanced cellular ingrowth and accelerated hydrolytic degradation of the scaffolds due to the incorporation of MNPs. The results also support the view that the degradation rate of the magnetic scaffolds is properly regulated to allow the ingrowth of cells and neoblood vessel formation, which consequently contributes to excellent tissue compatibility of the magnetic scaffolds, enabling feasible use as implantable scaffolds. Subsequent tests performed in the rat segmental defects using the representative 15MNP magnetic scaffolds rolled in a cylindrical form proved a significant level of neobone formation in the defect sites, with more compact and better integrated form with the edge of host bone, while exhibiting a faster degradation sign of scaffold than the pure PCL (similar to a result in subcutaneous tissue), furthermore, active cellular responses from the adjacent ulna surface to the magnetic scaffold and the consequent formation of bone matrix in the space apparently supported active bone regenerative potential of the magnetic nanofiber scaffolds.

Both *in vitro* and *in vivo* tests confirmed excellent biocompatibility and the potential usefulness of the MNP-incorporated magnetic nanofiber scaffolds for tissue regeneration and engineering particularly for bone. At this point, those improved cellular and tissue reactions to the magnetic nanofiber scaffolds need to be reasoned. It is first considered that the improved hydrophilicity of magnetic scaffolds would be acknowledged in part. However, not only the altered hydrophilic nature, but other properties of the nanofiber scaffolds such as magnetic properties are possibly reasoned for this. Some recent studies have reported the beneficial effects of the superparamagnetic nanoparticles within biomaterials such as calcium phosphate bioceramics, poly(lactic-co-glycolic) scaffolds, and hydroxyapatite-collagen scaffolds on bone cell proliferation and differentiation *in vitro* and/or bone formation *in vivo* [10], [21], [22], [43]–[46]. The HA-magnetite ceramic composite scaffolds sintered at high temperature showed significantly enhanced osteoblastic cell behaviors under static magnetic fields with respect to HA counterpart scaffold, particularly at the early phase of cell proliferation, and also exhibited in vivo tissue compatibility, which was suggested as the magnetic scaffold fixation and magnetic drug delivery [21]. The calcium phosphate scaffolds incorporated with MNPs have shown to promote the proliferation and differentiation of osteosarcoma cell line [43]. For the polymer magnetic nanocomposites, the PLGA scaffolds incorporated with MNPs showed increased cell proliferation with alteration in cell cycle although no significant improvement was noticed in the differentiation of osteoblastic cell line [22]. Not only on the osteoblastic cells, but also on the myoblasts, the effects of MNPs-incorporation within PLGA scaffolds have also been demonstrated in the cellular proliferation and migration [44]. Although these series of works on the magnetic scaffolds have reported significantly improved cell proliferation and sometimes stimulated differentiation, there has been no study to elucidate possible mechanisms using scaffold systems. On the other hand, it was suggested that each magnetic nanoparticle could be considered as a single magnetic domain on a nanoscale, which might affect the ion channels of cell membrane so as to influence the cell behaviors [45], [46]. Despite the intensity of the nanoscale-generated magnetic field being extremely low, the total effect would likely be strengthened with an increased amount of MNPs, thus it would have a stronger influence on the cell responses. Induced magnetic fields possibly generated at the microenvironmental levels *in vitro* and *in vivo* could be considered as another sort of physical factors that influencing surrounding cellular responses.

The magnetic scaffolds are considered to play effective roles as a fixed implantable system that provides *in-situ* magnetism, enabling adjustment of the scaffold activity to the patient's personal need, which largely overcomes the current difficulties in magnetic guiding devices [47]–[49]. In this manner, although the current study investigated the static effects of the magnetic scaffolds on bone cell responses *in vitro* and bone formation *in vivo*, it would be an interesting study to see the magnetism-induced effects on those biological responses by applying external magnetic fields to the scaffolds loaded with cells for magnetism-induced tissue engineering or to the directly-implanted sites. This work could possibly clarify the role of magnetic fields induced by the scaffolds on such improvements in bone cell/tissue reactions. Furthermore, when applied alternating magnetic fields, the magnetism-induced heat is generated and this should alter the cellular status more intensely, allowing the possible hyperthermia treatments for bone-related diseases, which also remain as further study.

## Conclusions

Collectively, the MNPs-incorporated PCL nanofiber scaffolds were proven to have some fascinating properties for the applications in bone regeneration, which include increased hydrophilicity, accelerated degradation and apatite forming ability, and the mechanical properties such as strength, elastic modulus, and elongation, while exhibiting MNP-related magnetic properties. Furthermore, biological potentials including excellent cellular interactions and osteogenesis *in vitro* as well as tissue compatibility and bone regenerative ability *in vivo* were demonstrated. Results provided herein are considered to open the door to a new class of bone regenerative materials, the magnetic nanofibrous scaffolds.

## References

[1] Hwang NS, Varghese S, Elisseeff J (2008) Controlled differentiation of stem cells. Adv Drug Deliv Rev 60: 199–214. 18006108 10.1016/j.addr.2007.08.036 PMC2712932

[2] Singh A, Elisseeff J (2010) Biomaterials for stem cell differentiation. J Mater Chem 20: 8832–8847.

[3] Patel JH, Nayyer L, Seifalian AM (2013) Chondrogenic potential of bone marrow-derived mesenchymal stem cells on a novel, auricular-shaped, nanocomposite scaffold. J Tissue Eng 4 2041731413516782. 10.1177/2041731413516782 PMC3927962 24555012

[4] Holzwarth JM, Ma PX (2011) Biomimetic nanofibrous scaffolds for bone tissue engineering. Biomaterials 32 ((36)) 9622–9629. 21944829 10.1016/j.biomaterials.2011.09.009 PMC3195926

[5] Revi D, Vineetha VP, Muhamed J, Rajan A, Anilkumar TV (2013) Porcine cholecyst–derived scaffold promotes full-thickness wound healing in rabbit. J Tissue Eng 4 2041731413518060. 10.1177/2041731413518060 PMC3927752 24555014

[6] Kim HJ, Park IH, Kim JH, Cho CS, Kim MS (2013) Gas Foaming Fabrication of Porous Biphasic Calcium Phosphate for Bone Regeneration. Tissue Eng Regen Med 9: 63–68.

[7] Jang JH, Castano O, Kim HW (2009) Electrospun materials as potential platforms for bone tissue engineering. Adv Drug Deliv Rev 61 ((12)) 1065–1083. 19646493 10.1016/j.addr.2009.07.008

[8] Langer R, Vacant JP (1993) Tissue eng. Science 260 ((5110)) 920–926. 8493529 10.1126/science.8493529

[9] Chung HJ, Park TG (2007) Surface engineered and drug releasing pre-fabricated scaffolds for tissue engineering. Adv Drug Deliv Rev 59 ((4–5)) 249–262. 17482310 10.1016/j.addr.2007.03.015

[10] Bock N, Riminucci A, Dionigi C, Russo A, Tampieri A, et al (2010) A novel route in bone tissue engineering: Magnetic biomimetic scaffolds. Acta Biomater 6: 786–796. 19788946 10.1016/j.actbio.2009.09.017

[11] Santis RD, Gloria A, Russo T, D'Amora U, Zeppetelli S, et al (2011) A basic approach toward the development of nanocomposite magnetic scaffolds for advanced bone tissue engineering. J Appl Poly Sci 122: 3599–3605.

[12] Lv G, He F, Wang X, Gao F, Zhang G, et al (2008) Novel nanocomposite of nano Fe_3_O_4_ and polylactide nanofibers for application in drug uptake and induction of cell death of leukemia cancer cells. Langmuir 24: 2151–2156. 18193905 10.1021/la702845s

[13] Lopez MB, Redondo YP, Santis RD, Gloria A, Ambrosio L, et al (2011) Poly(caprolactone) based magnetic scaffolds for bone tissue engineering. J Appl Phys 109: 07B313.

[14] Kannarkat JT, Battogtokh J, Philip J, Wilson OC, Mehl PM (2010) Embedding of magnetic nanoparticles in polycaprolactone nanofiber scaffolds to facilitate bone healing and regeneration. J Appl Phys 107: 09B307.

[15] Tampieri A, Landi E, Valentini F, Sandri M, D'Alessandro T, et al (2011) A conceptually new type of bio-hybrid scaffold for bone regeneration. Nanotechnol 22: 015104. 10.1088/0957-4484/22/1/015104 21135464

[16] Lang SB (1996) Pyroelectric effect in bone and tendon. Nature 212: 704–705.

[17] Nomura S, Yamamoto TT (2000) Molecular events caused by mechanical stress in bone. Matrix Biol 19: 91–96. 10842092 10.1016/s0945-053x(00)00050-0

[18] Hadjipanayis CG, Bonder MJ, Balakrishnan S, Wang X, Mao H, et al (2008) Metallic iron nanoparticles for MRI contrast enhancement and local hyperthermia. Small 4: 1925–1929. 18752211 10.1002/smll.200800261 PMC2709953

[19] Campbell RB (2007) Battling tumors with magnetic nanotherapeutics and hyperthermia: turning up the heat. Nanomedicine 2: 649–652. 17976026 10.2217/17435889.2.5.649

[20] Kumar CSSR, Mohammad F (2011) Magnetic nanomaterials for hyperthermia-based therapy and controlled drug delivery. Adv Drug Deliv Rev 63 ((9)) 789–808. 21447363 10.1016/j.addr.2011.03.008 PMC3138885

[21] Panseri S, Cunha C, D'Alessandro T, Sandri M, Russo A, et al (2012) Magnetic hydroxyapatite bone substitutes to enhance tissue regeneration: Evaluation in vitro using osteoblast-like cells and in vivo in a bone defect. PLoS One 7 ((6)) e38710. 22685602 10.1371/journal.pone.0038710 PMC3369900

[22] Lai K, Jiang W, Tang JZ, Wu Y, He B, et al (2012) Superparamagnetic nano-composite scaffolds for promoting bone cell proliferation and defect reparation without a magnetic field. RSC Adv 2: 13007–13017.

[23] Shin SH, Purevdorj O, Castano O, Planell JA, Kim HW (2012) A short review: Recent advances in electrospinning for bone tissue regeneration. J Tissue Eng doi:10.1177/2041731412443530 10.1177/2041731412443530 PMC3324843 22511995

[24] Lim SH, Mao HQ (2009) Electrospun scaffolds for stem cell engineering. Adv Drug Deliv Rev 61 ((12)) 1084–1096. 19647024 10.1016/j.addr.2009.07.011

[25] Gunatillake PA, Adhikari R (2003) Biodegradable synthetic polymers for tissue engineering. Eur Cells Mater 5: 1–16. 10.22203/ecm.v005a01 14562275

[26] Lee JJ, Yu HS, Hong SJ, Jeong I, Jang JH, et al (2009) Nanofibrous membrane of collagen–polycaprolactone for cell growth and tissue regeneration. J Mat Sci: Mat Med 20: 1927–35. 10.1007/s10856-009-3743-z 19365614

[27] Singh RK, Kim TH, Patel KD, Knowles JC, Kim HW (2012) Biocompatible magnetite nanaoparticles with varying silica coating layer for use in biomedicine: physicochemical and magnetic properties and cellular compatibility. J Biomed Mater Res A 100: 1734–42. 22447364 10.1002/jbm.a.34140

[28] Yu HS, Jang JH, Kim TI, Lee HH, Kim HW (2009) Apatite Mineralized polycaprolactone nanofibrous web as a bone tissue regeneration substrate. J Biomed Mater Res A 88A: 747–54. 10.1002/jbm.a.31709 18357562

[29] Oh SA, Kim SH, Won JE, Kim JJ, Shin US, et al (2010) Effects on growth and osteogenic differentiation of mesenchymal stem cells by the zinc-added sol-gel bioactive glass granules. J Tissue Eng 2010: 475260. 10.4061/2010/475260 PMC3040507 21350651

[30] Hammond C (1997) The Basics of Crystallography and Diffraction (Oxford:Oxford University Press).

[31] Cabaco MI, Besnard M, Danten Y, Coutinho JAP (2011) Solubility of CO_2_ in 1-butyl-3-methyl-imidazolium-trifluoro Acetate Ionic Liquid Studied by Raman Spectroscopy and DFT Investigations. J Phys Chem B 115: 3538–3550. 21410211 10.1021/jp111453a

[32] Zhang F, Wang CC (2008) Fabrication of one-dimensional iron oxide/silica nanostructures with high magnetic sensitivity by dipole-directed self-assembly. J Phys Chem C 112: 15151–15156.

[33] Meng J, Zhang Y, Qi X, Kong H, Wang C, et al (2010) Paramagnetic nanofibrous composite films enhance the osteogenic responses of pre-osteoblast cells. Nanoscale 2: 2565–2569. 20949222 10.1039/c0nr00178c

[34] Houa R, Zhanga G, Dua G, Zhanb D, Congb Y, et al (2013) Magnetic nanohydroxyapatite/PVA composite hydrogels for promoted osteoblast adhesion and proliferation. Coll Surf B: Biointerfac 103: 318–325. 10.1016/j.colsurfb.2012.10.067 23261554

[35] Lin TC, Lin FH, Lin JC (2012) In vitro feasibility study of the use of a magnetic electrospun chitosan nanofiber composite for hyperthermia treatment of tumor cells. Acta Biomater 8: 2704–2711. 22484694 10.1016/j.actbio.2012.03.045

[36] Meenach SA, Hilt JZ, Anderson KW (2010) Poly(ethylene glycol)-based magnetic hydrogel nanocomposites for hyperthermia cancer therapy. Acta Biomater 6: 1039–1046. 19840875 10.1016/j.actbio.2009.10.017

[37] Singh RK, El-Fiqi AM, Patel KD, Kim HW (2012) A novel preparation of magnetic hydroxyapatite nanotubes. Mater Lett 75: 130–133.

[38] Mavis B, Demirtas TT, Gumusderelioglu M, Gunduz G, Colak U (2009) Synthesis, characterization and osteoblastic activity of polycaprolactone nanofibers coated with biomimetic calcium phosphate. Acta Biomater 5: 3098–3111. 19426840 10.1016/j.actbio.2009.04.037

[39] Tanahashi M, Matsuda T (1997) Surface functional group dependence on apatite formation on self-assembled monolayers in a simulated body fluid. J Biomed Mater Res A 34: 305–315. 10.1002/(sici)1097-4636(19970305)34:3<305::aid-jbm5>3.0.co;2-o 9086400

[40] Lee EJ, Teng SH, Jang TS, Wang P, Yook SW, et al (2010) Nanostructured poly(α-caprolactone)–silica xerogel fibrous membrane for guided bone regeneration. Acta Biomater 6: 3557–3565. 20304111 10.1016/j.actbio.2010.03.022

[41] Kim HW, Song JH, Kim HE (2005) Nanofiber generation of gelatin- hydroxyapatite biomimetics for guided tissue regeneration. Adv Funct Mater 15: 1988–1994.

[42] Presa PDL, Luengo Y, Multigner M, Costo R, Morales MP, et al (2012) Study of Heating Efficiency as a Function of Concentration, Size, and Applied Field in γ-Fe_2_O_3_ Nanoparticles. J Phys Chem C 116 ((48)) 25602–25610.

[43] Gu ZW, Wu Y, Jiang W, Wen XT, He B, et al (2010) A novel calcium phosphate ceramic-magnetic nanoparticle composite as a potential bone substitute. Biomed Mater 5: 015001. 10.1088/1748-6041/5/1/015001 20057017

[44] Hu H, Jiang W, Lan F, Zeng X, Ma S, et al (2013) Synergic effect of magnetic nanoparticles on the electrospun aligned superparamagnetic nanofibers as a potential tissue engineering scaffold. RSC Adv 3: 879–86.

[45] Hughes S, EI Haj AJ, Dobson J (2005) Magnetic micro- and nanoparticle mediated activation of mechanosensitive ion channels. Med Eng Phys 27 ((9)) 754–762. 15985383 10.1016/j.medengphy.2005.04.006

[46] Huang DM, Hsiao JK, Chen YC, Chien LY, Yao M, et al (2009) The promotion of human mesenchymal stem cell proliferation by superparamagnetic iron oxide nanoparticles. Biomaterials 30 ((22)) 3645–3651. 19359036 10.1016/j.biomaterials.2009.03.032

[47] Wei Y, Zhang X, Song Y, Han B, Hu X, et al (2011) Magnetic biodegradable Fe_3_O_4_/CS/PVA nanofibrous membranes for bone regeneration. Biomed Mater 6 ((5)) 055008. 21893702 10.1088/1748-6041/6/5/055008

[48] Wu C, Fan W, Zhu Y, Gelinsky M, Chang J, et al (2011) Multifunctional magnetic mesoporous bioactive glass scaffolds with a hierarchical pore structure. Acta Biomater 7 ((10)) 3563–3572. 21745610 10.1016/j.actbio.2011.06.028

[49] Zeng XB, Hu H, Xie LQ, Lan F, Jiang W, et al (2012) Magnetic responsive hydroxyapatite composite scaffolds construction for bone defect reparation. Inter J Nanomed 7: 3365–3378. 10.2147/IJN.S32264 PMC3405892 22848165

